# Skin Diseases: From Molecular Mechanisms to Novel Therapeutic Approaches—An Editorial

**DOI:** 10.3390/biomedicines14102315

**Published:** 2026-10-12

**Authors:** Zhiqin Zhang, Can Gong, Guangtong Deng

**Affiliations:** 1 Department of Dermatology, Xiangya Hospital, Central South University, 87 Xiangya Road, Changsha 410008, China; zhangzhiqin1006@126.com (Z.Z.); gongcan021206@163.com (C.G.); 2 National Engineering Research Center of Personalized Diagnostic and Therapeutic Technology, Changsha 410008, China; 3 Hunan Engineering Research Center of Skin Health and Disease, Central South University, Changsha 410008, China; 4 Hunan Key Laboratory of Skin Cancer and Psoriasis, Central South University, Changsha 410008, China; 5 National Clinical Research Center of Geriatric Disorders, Xiangya Hospital, Central South University, Changsha 410008, China; 6 Hunan Key Laboratory of Aging Biology, Changsha 410008, China

The skin serves as the body’s largest and most exposed organ, making it uniquely vulnerable to a wide array of external insults and internal dysregulations [1]. Skin diseases affect approximately 4.7 to 4.9 billion individuals worldwide [2], ranking among the foremost causes of disease burden and accounting for 1.79% of the global disability-adjusted life-year burden [3]. Together, these epidemiological and clinical factors position dermatological conditions as a central global public health priority.

Over the past few decades, rapid advances across basic, translational, and clinical research have dramatically reshaped our understanding of dermatological pathogenesis, thereby accelerating the discovery of novel therapeutic targets. Despite these encouraging developments, significant knowledge gaps persist regarding the intricate mechanisms that drive disease initiation, progression, and therapeutic resistance, and many current interventions remain suboptimal in terms of efficacy or safety. It is against this backdrop that this Special Issue was conceived, bringing together six manuscripts that span a broad spectrum of disease entities—from immune-mediated inflammatory conditions (such as psoriasis and atopic dermatitis (AD)) to autoimmune depigmentation disorders (like vitiligo), rare genodermatoses, and chronic genital dermatoses—all united by a common focus on elucidating targetable molecular mechanisms. Below, we synthesize the key findings and implications of the articles assembled in this Special Issue.

Immune-mediated inflammatory skin diseases are among the most prevalent and burdensome categories of dermatological conditions, with psoriasis and AD standing out as the two most common forms [4]. Three contributions in this Special Issue focus on these two diseases, advancing our understanding of their pathogenesis and the corresponding therapeutic strategies. Jin et al. [5] demonstrated that hyper-pyroptotic monocyte-derived macrophages drive psoriasis progression via CXCL16-mediated Th17 activation, identifying pyroptosis and the CXCL16-CXCR6 axis as promising therapeutic targets. Huang et al. [6] showed that mesenchymal stem cell (MSC)-derived exosomes significantly reduce epidermal thickness and inflammatory cytokine levels in imiquimod-induced-psoriasis-like mice, with umbilical-cord-derived exosomes showing superior efficacy. Zuo et al. [7] showed that JAK1 inhibition specifically attenuates mechanical alloknesis in atopic dermatitis independent of its classical anti-inflammatory or barrier-restoring properties. Collectively, these three studies illustrate the trajectory from mechanistic discovery to therapeutic innovation, exemplifying the translational focus of this Special Issue.

Beyond these common inflammatory conditions, three articles in this Special Issue address rare, localized, or complex dermatological phenotypes, shedding light on their unique molecular signatures and diagnostic pathways. Poffet et al. [8] revealed distinct molecular signatures distinguishing genital lichen sclerosus (LS) from lichen planus genitalis (Lpg)-LS with CCL27/MARCO upregulation and Lpg with IL-1 pathway and B-cell dominance, providing a rationale for disease-specific therapy. Liu et al. [9] reported half-siblings with KRT10-mutated bullous congenital ichthyosiform erythroderma complicated by a *Trichophyton rubrum* infection, highlighting both infection susceptibility in ichthyosis and the utility of blocker displacement amplification (BDA) for detecting mosaic mutations. Marrapodi, Marini, and Bellei [10] comprehensively reviewed vitiligo pathogenesis, concluding that oxidative stress, metabolic dysfunction, senescence, and multiple cell-death pathways converge on melanocyte destruction and that targeting these upstream events may offer a more durable benefit than immunosuppression alone.

The articles in this Special Issue collectively demonstrate that dermatological diseases arise from interconnected molecular networks involving immune dysregulation, cell death, metabolic dysfunction, and genetic predisposition. Several unifying themes emerge: multi-omics integration enables dissection of disease heterogeneity; molecular profiling can distinguish clinically overlapping conditions to guide precision treatment; upstream therapeutic targeting—addressing initiating events rather than downstream inflammation—offers durable benefits; and novel platforms such as MSC-derived exosomes and BDA-based diagnostics are expanding the therapeutic and diagnostic repertoire.

Looking forward, we can note several priorities that merit attention. First, prospective, large-scale cohorts are urgently needed to validate biomarkers for predicting treatment response and disease recurrence, as high-resolution molecular profiling can be used to differentiate phenotypically similar skin diseases to inform personalized treatment strategies [11,12]. Multi-omics integration—spanning genomics, transcriptomics, proteomics, and metabolomics—has proven invaluable for dissecting disease heterogeneity and identifying novel biomarkers [13,14,15]. Furthermore, the efficacy of upstream-targeting strategies—alone and in combination with immunomodulators—must be systematically evaluated. The identification of shared pathways across diseases introduces the possibility of “one-drug-multiple-disease” approaches [16], potentially streamlining drug development. Ultimately, the success of precision dermatology will depend not only on scientific discovery but also on the implementation of individualized care and vigilance with respect to adverse events associated with emerging targeted therapies.

The Guest Editors sincerely thank all authors, reviewers, and Editorial Board Members for their invaluable contributions. We hope this Special Issue inspires novel research and accelerates the translation of mechanistic insights into improved patient care.
